# Stanozolol and Danazol Have Different Effects on Hematopoiesis in the Murine Model of Immune-Mediated Bone Marrow Failure

**DOI:** 10.3389/fmed.2021.615195

**Published:** 2021-05-28

**Authors:** Hongmin Li, Zhangbiao Long, Tao Wang, Bing Han

**Affiliations:** ^1^Department of Hematology, Peking Union Medical College Hospital (PUMCH), Chinese Academy of Medical Science, Beijing, China; ^2^Institute of Basic Medical Sciences, Peking Union Medical College, Chinese Academy of Medical Sciences, Beijing, China

**Keywords:** stanozolol, danazol, aplastic anemia, hematopoiesis, animal model

## Abstract

**Background:** Stanozolol and danazol are widely used in the treatment of aplastic anemia; however, their mechanisms of action are unclear.

**Methods:** Bone marrow mononuclear cells from 10 patients newly diagnosed with aplastic anemia and 10 healthy volunteers were collected and cultured together with stanozolol, danazol, or blank control separately for marrow colony assays. K562 cell lines that had been incubated with stanozolol, danazol, or blank control were tested for erythroid or megakaryocytic differentiation. Meanwhile, CB6F1/Crl mice were injected with 1 × 10^6^ C57BL/6 donor-originated lymphocytes after irradiation with 5 Gy total body irradiation to establish a model for immune-mediated bone marrow failure (aplastic anemia mouse model). Mice with aplastic anemia were treated with cyclosporin A monotherapy, cyclosporin A in combination with stanozolol, and cyclosporin A in combination with danazol for 30 days. Peripheral blood cell counts once a week and bone marrow colony assays at the end of 1 month were performed. The proportion of T cell subsets, level of inflammatory factors, erythropoietin, and thrombopoietin were detected before and after treatment. The levels of erythropoietin receptors on bone marrow mononuclear cells after treatment were tested using western blotting.

**Results:** In the *ex vivo* experiments, the number of burst-forming units-erythroid; colony-forming units-granulocyte and macrophage; and colony-forming units-granulocyte, erythrocyte, monocyte, and megakaryocyte in the patients with aplastic anemia were significantly lower than that in the normal controls (*P* < 0.05). However, the number of colonies and mean fluorescence intensity of CD235a or CD41 expression in the harvested cultured cells were not significantly different among the different treatment groups in the patients with aplastic anemia, normal controls, and K562 cell lines. These results show that stanozolol and danazol produce no direct hematopoiesis-stimulating effects on progenitor cells. In the *in vivo* experiment, the mice with aplastic anemia treated with cyclosporin A and danazol exhibited the most rapid recovery of platelet; the platelet count returned to normal levels after 3 weeks of treatment, which was at least 1 week earlier than in the other groups. In contrast, mice treated with cyclosporin A and stanozolol exhibited the highest hemoglobin level at the end of treatment (*P* < 0.05). Bone marrow colony assays at 30 days showed that the number of burst-forming units-erythroid was the highest in mice treated with cyclosporin A and stanozolol, while the number of colony-forming units-granulocyte and macrophage was the highest in those treated with cyclosporin A and danazol. Compared to cyclosporin A monotherapy, additional stanozolol and danazol can both increase the level of regulatory T cells and upregulate interleukin-10, inhibiting the expression of tumor necrosis factor-α (*P* < 0.05). However, IL-2 was more effectively reduced by danazol than by stanozolol (*P* < 0.05). The cyclosporin A- and stanozolol-treated mice showed higher serum erythropoietin (corrected by hemoglobin level) and higher erythropoietin receptor levels in bone marrow mononuclear cells than the other groups (*P* < 0.05).

**Conclusions:** Neither stanozolol nor danazol directly stimulated hematopoiesis *in vitro*. However, *in vivo*, stanozolol may exhibit an advantage in improving erythropoiesis, while danazol may induce stronger effects on platelets. Both danazol and stanozolol exhibited immunosuppressive roles. Stanozolol could enhance the secretion of erythropoietin and expression of erythropoietin receptor in bone marrow mononuclear cells.

## Introduction

Aplastic anemia (AA) is a syndrome associated with hematopoietic failure owing to various reasons, which leads to diminished or no hematopoietic precursors in the bone marrow and attendant single or multi-lineage cytopenia. Based on the severity of the disease, AA can be classified as severe AA (SAA) or non-severe AA (NSAA). Patients with SAA are characterized by acute onset, rapid disease progression, and high mortality. In terms of therapeutic approaches, allogeneic hematopoietic stem cell transplantation (HSCT) or intensive immunosuppressive treatment based on cyclosporin A (CsA) in combination with antithymocyte globulin is considered as the first-line treatment (1). For transfusion-dependent NSAA, antithymocyte globulin+cyclosporine A can also serve as the first-line therapy. However, in the real world, patients with NSAA are often treated with CsA alone owing to high costs and potential risks of antithymocyte globulin, especially in developing countries.

Androgens are frequently used in combination with CsA or sometimes as the only therapy for patients with NSAA. As a synthetic androgenic drug with weak masculinization side effects, danazol is used worldwide. It has been reported that the overall response rate of danazol monotherapy can be up to 40% in patients with AA. Particularly, danazol may help improve the level of platelets (PLT) (2–4). Stanozolol, another synthetic androgenic drug with a protein assimilation effect 30 times but an androgenic activity only 1/4 that of testosterone, has been widely used in China for years, especially when anemia is the dominant manifestation (5). Stanozolol is unavailable in countries other than China; studies focusing on stanozolol are limited and its underlying mechanism of action remains largely unknown.

In this study, the effects of stanozolol and danazol on hematopoiesis were investigated *in vitro* and *in vivo* using colony culture of hematopoietic cells and an immune-mediated AA mouse model. Through these experiments, we attempted to illustrate the difference between the two drugs with respect to their hematopoietic stimulation, immune-regulation effects, and pathways of action.

## Materials and Methods

### Bone Marrow Mononuclear Cell Colony Assay

Bone marrow samples were collected from 10 patients newly diagnosed with acquired AA and 10 age- and sex-matched healthy volunteers at Peking Union Medical College Hospital from January 2017 to January 2018. Written informed consent was obtained from all patients and healthy volunteers before sample collection and all procedures were approved by the ethics committee of Peking Union Medical Colleague Hospital. BMMNCs were isolated and inoculated into plates containing methylcellulose complete medium (MethoCult® #04434, STEMCELLTechnology Inc., Canada) at a density of 2 × 10^4^/ml with 10^−8^ M stanozolol, 10^−8^ M danazol, and an equivalent volume of solvent (as the negative control) and incubated at 37°C in an incubator containing 5 % CO_2_ for 12 days. The numbers of burst-forming units-erythroid (BFU-E); colony-forming units-granulocyte and macrophage (CFU-GM); and colony-forming units-granulocyte, erythrocyte, monocyte, and megakaryocyte (CFU-GEMM) were counted using an inverted microscope.

### Effects of Stanozolol and Danazol on the Expression of CD235a and CD41 and Hemoglobin Production in K562 Cells

The human chronic myeloid leukemia K562 cell line was cultured in plates containing RPMI 1,640 medium with 10% fetal bovine serum (FBS) at 37°C in 5% CO_2_. Cells undergoing logarithmic growth (1 × 10^6^/ml) were further inoculated into four culture flasks containing 3 ml of RPMI 1,640 complete medium with 1 × 10^−8^ mol/L stanozolol, 1 × 10^−8^ mol/L danazol, an equivalent volume of phosphate-buffered saline (negative control), and 25 μmol/L hemin or 5 nmol/L phorbol 12-myristate 13-acetate (positive controls) (6, 7), respectively. Each test was repeated in three independent flasks. The cells were collected after incubation for 24, 48, 72, and 96 h; centrifuged; and tested for the expression of CD235a and CD41 on the cell surface by a FACSanto II flow cytometer (Becton Dickinson, San Jose, CA)., with the following antibodies was used: phycoerythrin (PE)-anti-human CD235a monoclonal antibody (PE) and fluorescein isothiocyanate (FITC)-anti-human CD41 monoclonal antibody (FITC) (eBioscience,USA). Meanwhile, the proportion of benzidine-positive cells was calculated following benzidine staining.

### Construction of the Immune-Mediated AA Mouse Model

Induction of immune-mediated AA mouse model was performed as previously reported (8). Lymph node cells from parental B6 donors were homogenized, washed, and filtered. Twenty CB6F1/Crl (first hybridized generation of female BALB/c and male C57BL/6) mice received 5 Gy total body irradiation and were injected with a single dose of 1 × 10^6^ B6 lymph node cells through the lateral tail vein 4 to 6 h after irradiation. The AA mice were assigned into four groups and treated with different regimens for 30 days, as shown in Table 1. The stanazolol and danazol doses used *in vivo* were converted from human equivalent dose based on body surface area (9). The human oral dose of stanazolol and danazol was 6 mg/d and 600 mg/d separately, to convert this dose to mg/kg.d and multiply it by 12.3, which was the mouse oral dose. Then, we convert the oral dose to the intravenous dose by a factor of 0.3. The standard weight of an adult was calculated as 70 kg and a CB6F1/Crl mouse as 20 g. All experimental operations on mice were in accordance with the Declaration of Helsinki and were approved by the ethics committee of Peking Union Medical Colleague Hospital.

**Table 1:** Treatment regimens for AA mice with different groups.

**Group no**.	**Number of mice**	**Treatment regimen (intraperitoneal injection)**
G1	5	CsA, 50 mg/kg/d, days 0~30
G2	5	CsA, 50 mg/kg/d, days 0~30+Stanozolol, 0.3 mg/kg/d, days 1~30
G3	5	CsA, 50 mg/kg/d, days 0~30+Danazol, 30 mg/kg/d, days 1~30
G4	5	Equivalent volume of saline (blank control)

*Five healthy CB6F1/Crl mice were considered as normal controls (G5)*.

### Parameter Detection in Immune-Mediated AA Mouse

#### Complete Blood Count

Blood was collected from the intraorbital venous plexus of mice on days 0, 7, 14, 21, and 28. HGB, white blood cells (WBC), and PLT were counted using a Sysmex XT-2000i hematology system. According to a previous report, cell counts lower than two standard deviations below the mean values for the controls indicated cytopenia (10).

#### Bone Marrow Colony Assay BMMNCs

Mice were sacrificed on day 30 using cervical dislocation. Bone marrow cells were harvested from the dissected tibia and femur. After centrifugation, the BMMNCs were plated in methylcellulose media containing recombinant human interleukin 6 and erythropoietin (EPO) or recombinant murine interleukin 3 and stem cell factor (MethoCult® #03434, STEMCELL Technology Inc. Canada). The cells were plated at a density of 2.0 × 10^4^/plate and the colonies were maintained at 37°C in an atmosphere of 5% CO_2_ in an incubator for 12 days. The number of bone marrow colonies was counted using an inverted microscope. Bone marrow cells in untreated mice (G4) were harvested when the mice were near death.

#### Flow Cytometry Detection of Treg Cells

Blood samples (300 ul) collected from AA mice and normal controls at day 30 were processed with red blood cell lysate. The cells isolated from PBMCs were stained with allophycocyanin (APC)- Cyanine7 -anti-mouse CD4, PE-Cyanine 7-anti-mouse CD8, APC-anti-human CD25 for 25 min at 4°C. Afterwards, fixation and permebilization of the cells were performed for Foxp3 staining as an intracellular marker. The fixed and permebilized cells were stained with PE-anti-mouse Foxp3 antibody for 25 min at 4°C. The cells were washed twice with 1 ml PBS and centrifuged at 300 × g for 5 min at room temperature. The percentage of the stained cells was measured by a FACSanto II flow cytometer (Becton Dickinson, San Jose, CA). The CD4^+^, CD25^+^, and Foxp3^+^ cells were considered as Tregs. The monoclonal antibodies related were bought from eBioscience, USA.

#### ELISA Detection of Serum Cytokines

The serum was separated from venous blood samples of mice except those in G4, which died quickly after transplantation. The expression of inflammatory factors, including INFγ, TNFα, IL-2, and IL-10, as well as the levels of EPO and thrombopoietin (TPO), were detected using an ELISA kit (Beyotime Biotechnology, China) according to the manufacturer's instructions.

#### Expression of Erythropoietin Receptor (EPOR) in Bone Marrow Mononuclear Cells by Western Blotting

On day 30, 1 × 10^7^ BMMNCs were collected by group and lysed with ultrasonication in RIPA cell lysis buffer (Beyotime Biotechnology, Haimen, China), supplemented with 1 mM PMSF. The lysates were clarified by centrifugation at 12,000 rpm for 15 min at 4°C. After denatured by boiling in loading buffer (Transgen Biotech, China), the protein was loaded and separated on 12% glycine SDS-PAGE gel, and transferred to polyvinylidene difluoride (PVDF) membranes (0.45 μm; Millipore, Bedford, MA). EPOR (CST, Rabbit pAb) and β-actin (CST, Rabbit mAb) were probed with specific primary antibodies and HRP-conjugated secondary anti-rabbit antibodies (CST, anti-rabbit-IgG). The immune-complex on the membrane was visualized using an automatic chemo-luminescence image analysis system (Tanon, Shanghai, China) with HRP substrate luminol reagent and peroxide solution (Millipore).

### Statistical Analysis

The SPSS 22.0 software was used for data processing and statistical analysis. Data was presented as means with standard deviation. Data for BM colonies, flow cytometry analysis, complete blood count and ELISA detections were analyzed by unpaired *t*-test or Spearman correlation coefficient for binary variables. For continuous variables of several independent groups, variance analysis or non-parametric Mann–Whitney *U* test were used as appropriate. Variations of statistical significance were further subjected to *post hoc* pairwise analysis by applying the Mann–Whitney *U* test and Bonferroni's correction. Since level of EPO and TPO was influenced by the level of hemoglobin and platelet count, a linear regression analysis was used. For the level of EPO, the relationship between EPO and HGB levels was established by log transformation of HGB concentration and comparing the slopes and y intercepts of regression line by covariance analysis. The actual HGB concentration was substituted into the above regression equation and the value obtained was defined as the expected EPO level. The change in EPO (delta EPO, ΔEPO, expected EPO—truly measured EPO) was considered as the effects from the drugs. Similar analysis was explored for the level of TPO. Data obtained from flow cytometry were analyzed using the FlowJo V10 software. GraphPad Prism statistical software (version 7.0) was used to draw figures and charts. Image J software was used to quantify the relative protein expression. *P* < 0.05 was considered statistically significant.

## Results

### Effects of Stanozolol and Danazol on Hematopoiesis

#### Effects of Stanozolol or Danazol on Bone Marrow Hematopoietic Colony Formation

**Figure 1:**
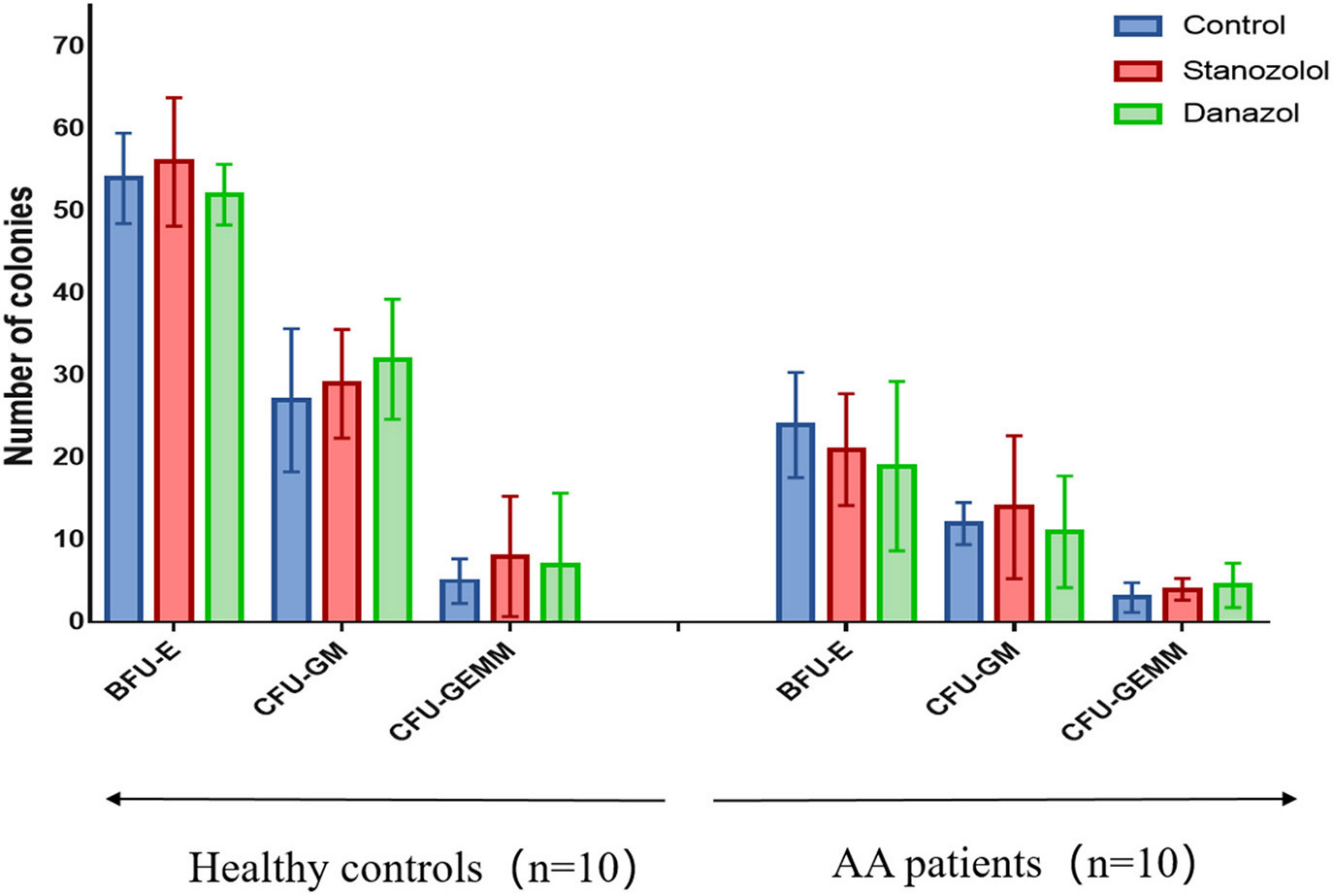
BMMNC colony assays in 10 healthy controls and 10 untreated NSAA patients. The number of BFU-E, CFU-GM, and CFU-GEMM colonies in NSAA patients was remarkably lower than that in healthy controls (*P* < 0.05), while the number of bone marrow colonies was not significantly different when incubated with stanozolol, danazol, or an equivalent volume of solvent. The histograms represent the average number colonies of 10 healthy controls and 10 NSAA patients. The error bars mean standard deviations. BFU-E, burst-forming units-erythroid (BFU-E); CFU-GM, colony-forming units-granulocyte and macrophage; CFU-GEMM, colony-forming units-granulocyte, erythrocyte, monocyte, and megakaryocyte.

There was no difference in the age and sex ratios between the 10 patients with newly diagnosed NSAA (six males and four females, average age: 43 ± 17 years) and the 10 healthy donors (five males and five females, average age: 42 ± 18 years, *P* > 0.05). The number of bone marrow colonies was significantly lower in patients with AA than that in the healthy subjects (BFU-E: 24 ± 7.60 vs. 54 ± 5.50; CFU-GM: 12 ± 2.55 vs. 27 ± 8.7; CFU-GEMM: 3 ± 1.8 vs. 5 ± 2.7, *P* < 0.05). However, upon incubation with stanozolol, danazol, or an equivalent volume of solvent, the number of colonies was not significantly different between the NSAA patients and healthy controls (*P* > 0.05, Figure 1). This indicates that neither stanozolol nor danazol can directly affect the growth and differentiation of hematopoietic stem and progenitor cells *in vitro*.

#### Effects of Stanozolol or Danazol on Erythroid and Megakaryocyte Differentiation in K562 Cells

**Figure 2:**
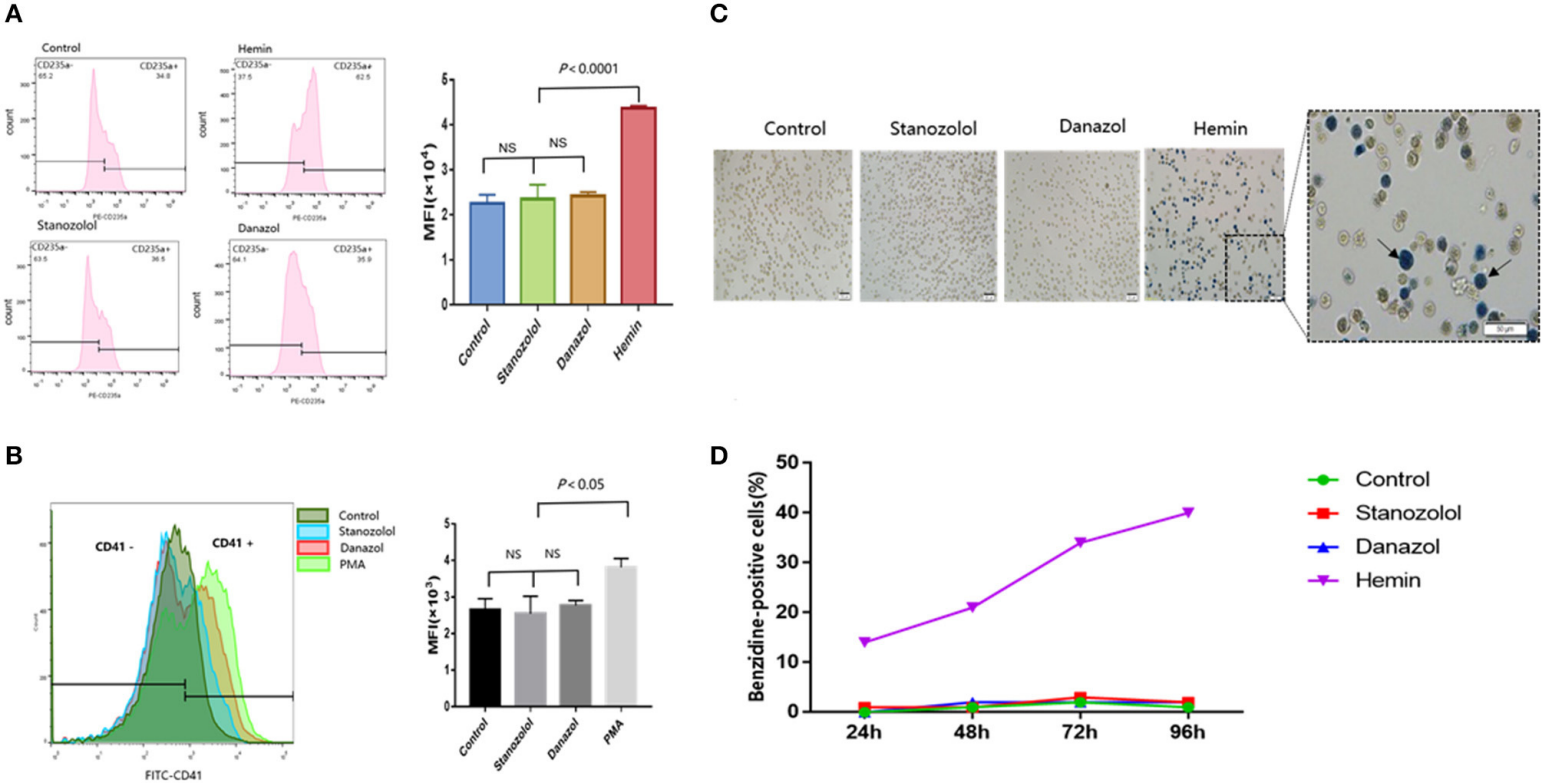
Effects of stanozolol and danazol on erythroid and megakaryocyte differentiation of K562 cells. **(A)** The expression of CD235a on the K562 cell surface after treatment with different drugs, as detected using flow cytometry. Each treatment group was repeated for 3 times, and the mean fluorescence intensity (MFI ± SD) was shown in the right chart. **(B)** The expression of CD41 on the K562 cell surface after treatment with different drugs, as assessed using flow cytometry. Each treatment group was repeated for 3 times, and the mean fluorescence intensity (MFI ± SD) was shown in the right chart. **(C)** Representation of cell morphology after 72 h of incubation with different treatments using microscopy; positive benzidine staining is indicated by arrows. **(D)** The rate of benzidine-positive cells following different treatments at different time points (count 100 cells).

Hemin was used as a positive control for erythroid differentiation, while phorbol 12-myristate 13-acetate served as a positive control for megakaryocyte differentiation. The expression of CD235a and CD41 in K562 cells showed no significant difference among cells treated with stanozolol, danazol, or an equivalent volume of solvent, as detected using flow cytometry (Figures 2A,B).

As shown in Figures 2C,D, the ratio of benzidine staining cells was not significantly different among the cells subjected to different treatment regimens at 24, 48, 72, and 96 h after incubation, indicating that stanozolol or danazol exhibits no direct erythroid differentiation effects *in vitro*.

### Construction of the Immune-Mediated AA Mouse Model

Preliminary results indicated that the untreated mice showed a marked decline in all three lineages on day 7, and all died within 15 days (blood was collected before death). Meanwhile, single drug treatment with stanozolol or danazol failed to prolong the survival of mice (data not shown).

#### Peripheral Complete Blood Cell Count

**Figure 3:**
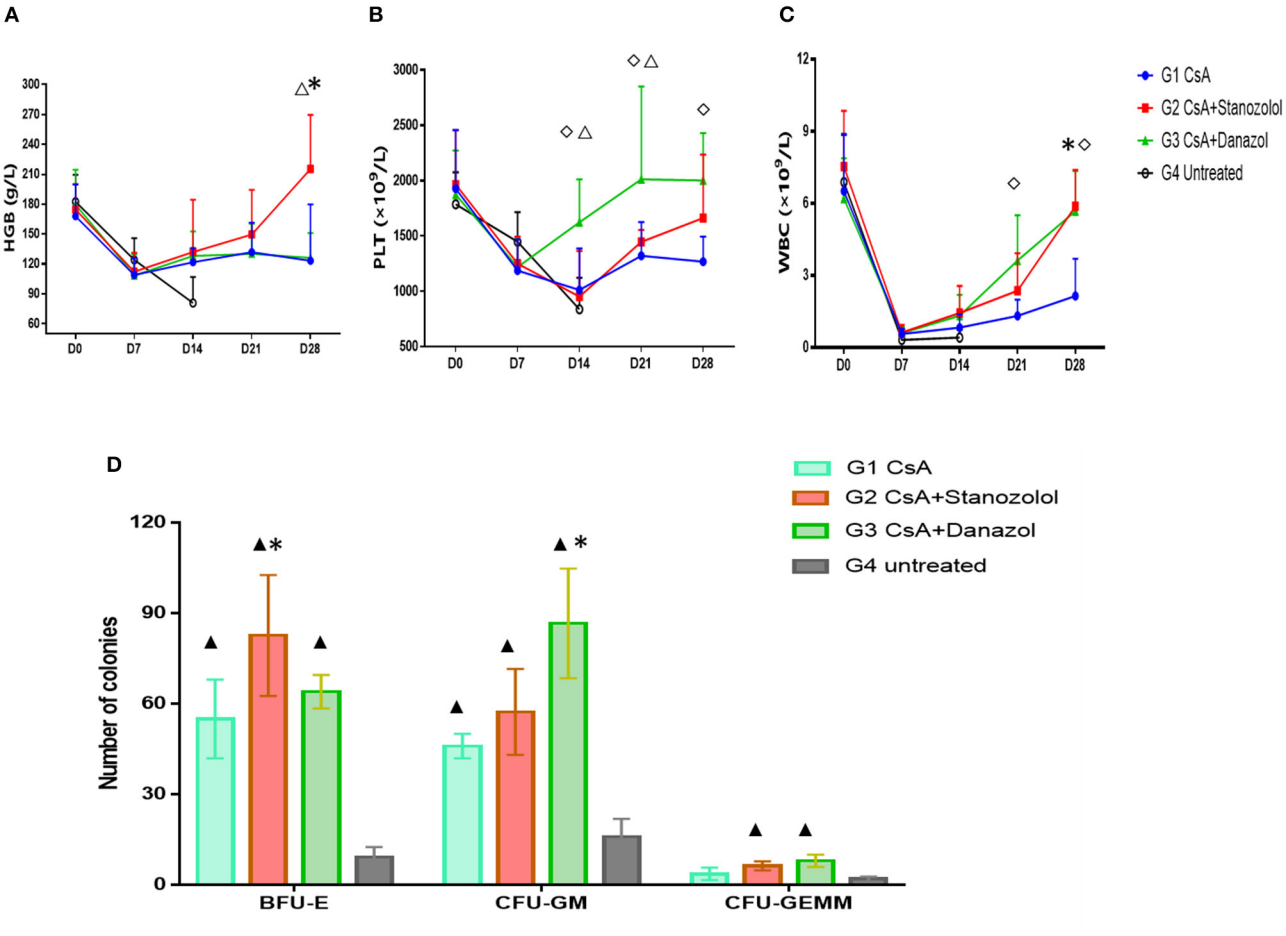
Hematopoiesis recovery in mice with different treatments. **(A–C)**. Hemoglobin (HGB), platelet (PLT), and white blood cell (WBC) levels in mice subjected to different treatments at different time points (represented as the means and SD). *indicates a significant difference between G2 and G1(*P* < 0.05); ♢ indicates a significant difference between G3 and G1 (*P* < 0.05); Δ indicates a significant difference between G2 and G3 groups (*P* < 0.05). **(D)** Colony numbers in different groups (*n* = 5) (on 2 × 10^4^ BMMNCs). Data was shown as means ± SD. *indicates a significant difference compared to the G1 group (*P* < 0.05) and ▴ indicates a significant difference compared to the G4 group (*P* < 0.05).

As shown in Figure 3, the number of blood cells in all mice reached the lowest points on day 7 after model generation and gradually recovered afterward. However, mice treated with CsA+stanozolol exhibited considerably higher HGB levels compared to mice treated with CsA+danazol and CsA alone on day 28 (*P* < 0.05, Figure 3A), whereas mice treated with CsA+danazol exhibited higher levels of PLT than the other two groups on day 14 (*P* < 0.05). The difference between CsA+danazol mice and CsA+stanozolol mice disappeared on day 28; however, it still existed between CsA+danazol mice and mice treated with CsA alone (Figure 3B).

Mice treated with CsA+danazol exhibited higher WBC levels than those treated with CsA alone on days 21 and 28; however, these values were not different from that in mice treated with CsA+stanozolol (Figure 3C).

These data suggested that compared to CsA monotherapy, both stanozolol and danazol could help in the recovery of hematopoiesis in the mouse model. Stanozolol produced an effect on erythroid hematopoiesis, while danazol may have been involved in megakaryopoiesis.

#### BMNC Colony Cultures in Different Treatment Groups

BMMNC colony culture on day 30 of modeling showed that the number of BFU-E was significantly higher in mice treated with CsA+stanozolol than in those treated with CsA alone (82 ± 57.3 vs. 55.0 ± 46.0, *P* = 0.01), whereas the number of CFU-GM was higher in mice treated with CsA+danazol than in those treated with CsA (86.7 ± 18.1 vs. 46 ± 4, *P* = 0.02). However, the number of BFU-E, CFU-GM, and CFU-GEMM was not significantly different between mice treated with CsA+stanozolol and CsA + danazol (Figure 3D).

### Immunoregulation Effects of Stanozolol and Danazol

#### Changes in T Cell Subsets

**Figure 4:**
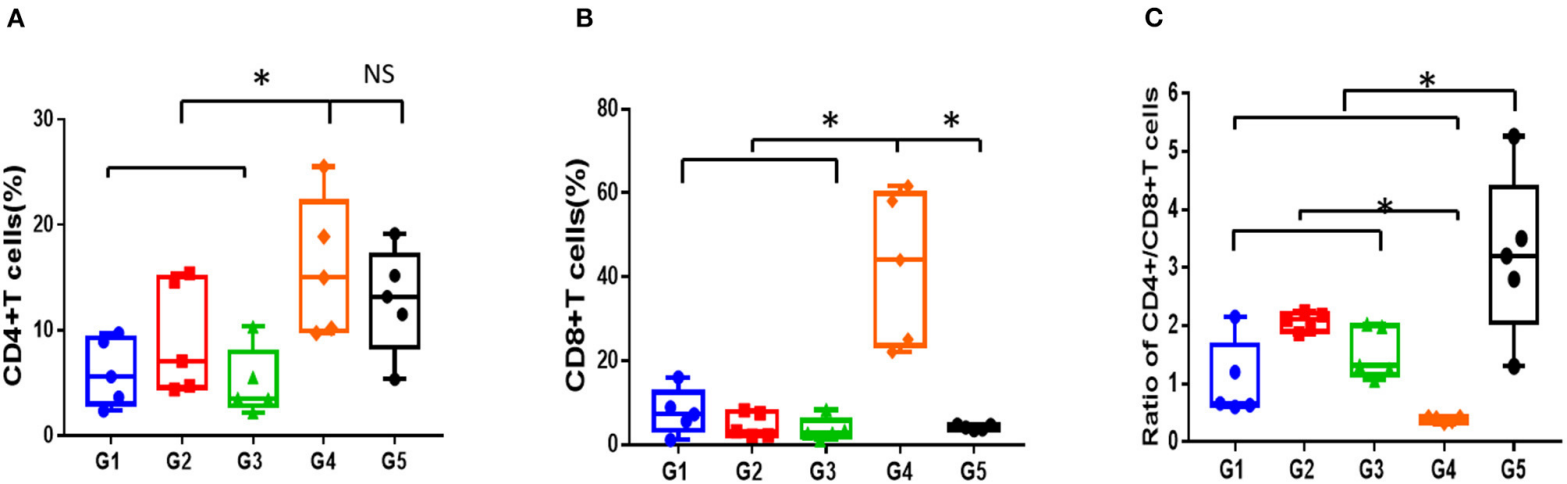
Proportion of T cell subsets in mice administered with different treatments, as detected using flow cytometry. **(A)** Proportion of CD4^+^T cells. **(B)** Proportion of CD8^+^T cells. **(C)** Ratio of CD4^+^/CD8+T cells. G1 CsA; G2 CsA+Stanozolol; G3 CsA+Danazol; G4 untreated; G5 normal control; ^*^indicates *P* < 0.05; NS indicates *P* > 0.05.

As is shown in Figures 4A–C, compared to normal mice, AA mice were infiltrated extensively by CD8^+^T cells in peripheral blood, with the proportion of CD4^+^/CD8^+^ T cells inverted. Meanwhile, the proportion of Treg/CD4^+^T cells in the mice with AA was significantly lower compared with that in the normal mice, which was consistent with the immunological features of human SAA (Supplementary Figures 1A,B). After 30 days of treatment, the proportion of CD4^+^/CD8^+^ T cells increased in all treatment groups; however, it remained lower than that in normal mice (*P* < 0.05). No statistical significance was found among the different treatment groups (Figure 4C). In contrast, the proportion of Treg cells in mice treated with CsA+stanozolol was similar to that in mice treated with CsA+danazol (7.49 ± 1.74 vs. 5.35 ± 1.6, *P* = 0.149) but was significantly higher than that in mice treated with CsA alone (*P* < 0.05, Supplementary Figures 1A,B).

#### Inflammation-Related Cytokines

**Figure 5:**
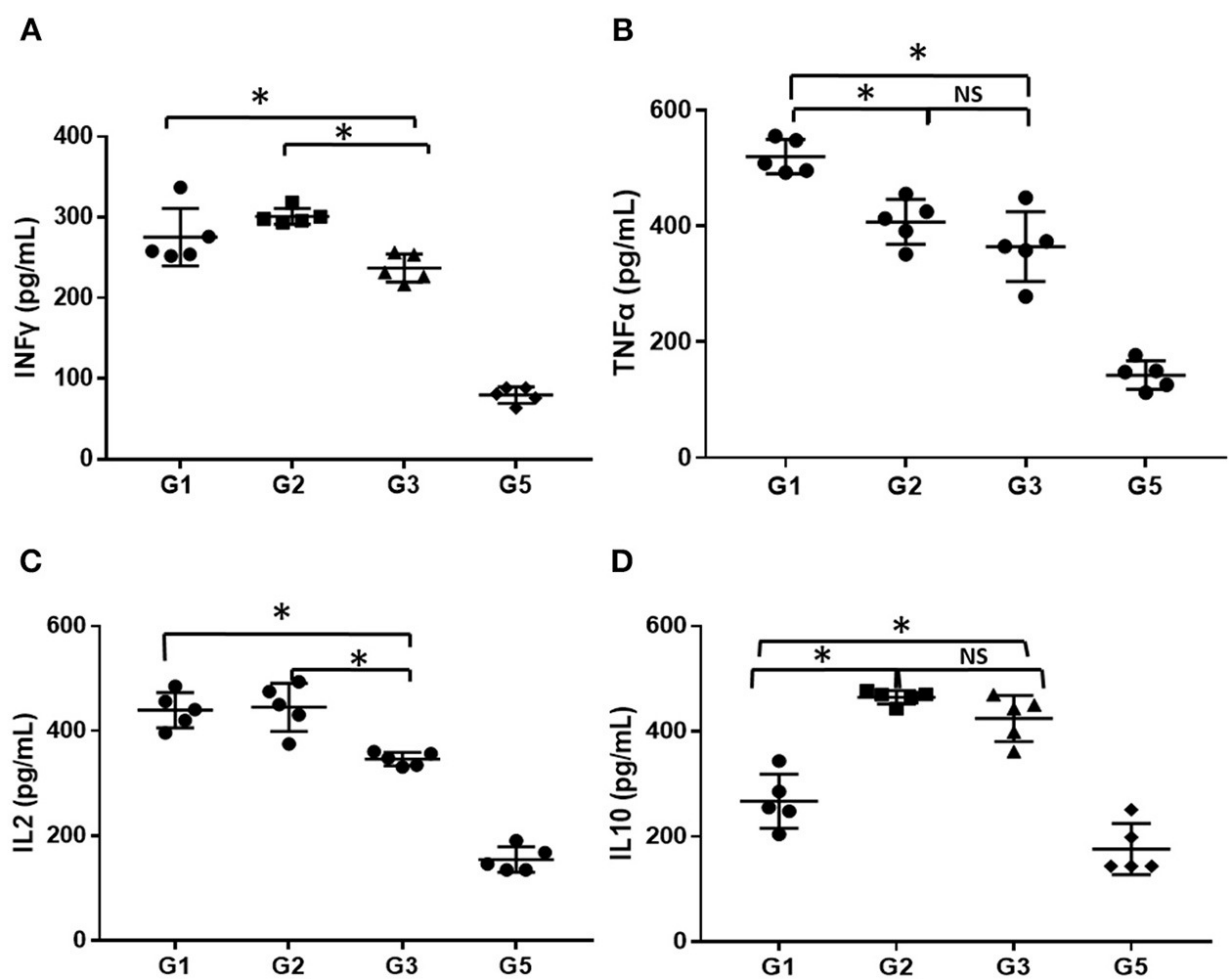
The level of inflammatory cytokines in mice administered different treatments. **(A)** The level of INFγ (pg/ml). **(B)** The level of TNFα (pg/ml). **(C)** The level of IL-2 (pg/ml). **(D)** The level of IL-10 (pg/ml). *indicates *P* < 0.05; NS indicates *P* > 0.05.

The serum levels of inflammatory factors (INFγ, TNFα, IL-2, and IL-10) in mice administered different treatments at 30 days was detected using ELISA and compared. As shown in Figure 5, the level of serum inflammatory factors was markedly higher in the AA mice than in the normal mice (*P* < 0.05), even treated with CsA alone or combined with either androgen. Mice treated with CsA+danazol exhibited lower levels of INFγ, TNFα, and IL-2 compared to mice treated with CsA alone. Meanwhile, mice treated with CsA+stanozolol exhibited lower levels of TNFα but similar levels of INFγ and IL-2 compared to mice treated with CsA alone. The level of IL-10 was upregulated in mice treated with CsA+ stanozolol and CsA+danazol compared to those treated with CsA alone. Collectively, these data showed that both danazol and stanozolol exhibited some immunosuppressive effects; however, the effects of danazol appeared to be more comprehensive.

#### Serum EPO and TPO Levels

**Figure 6:**
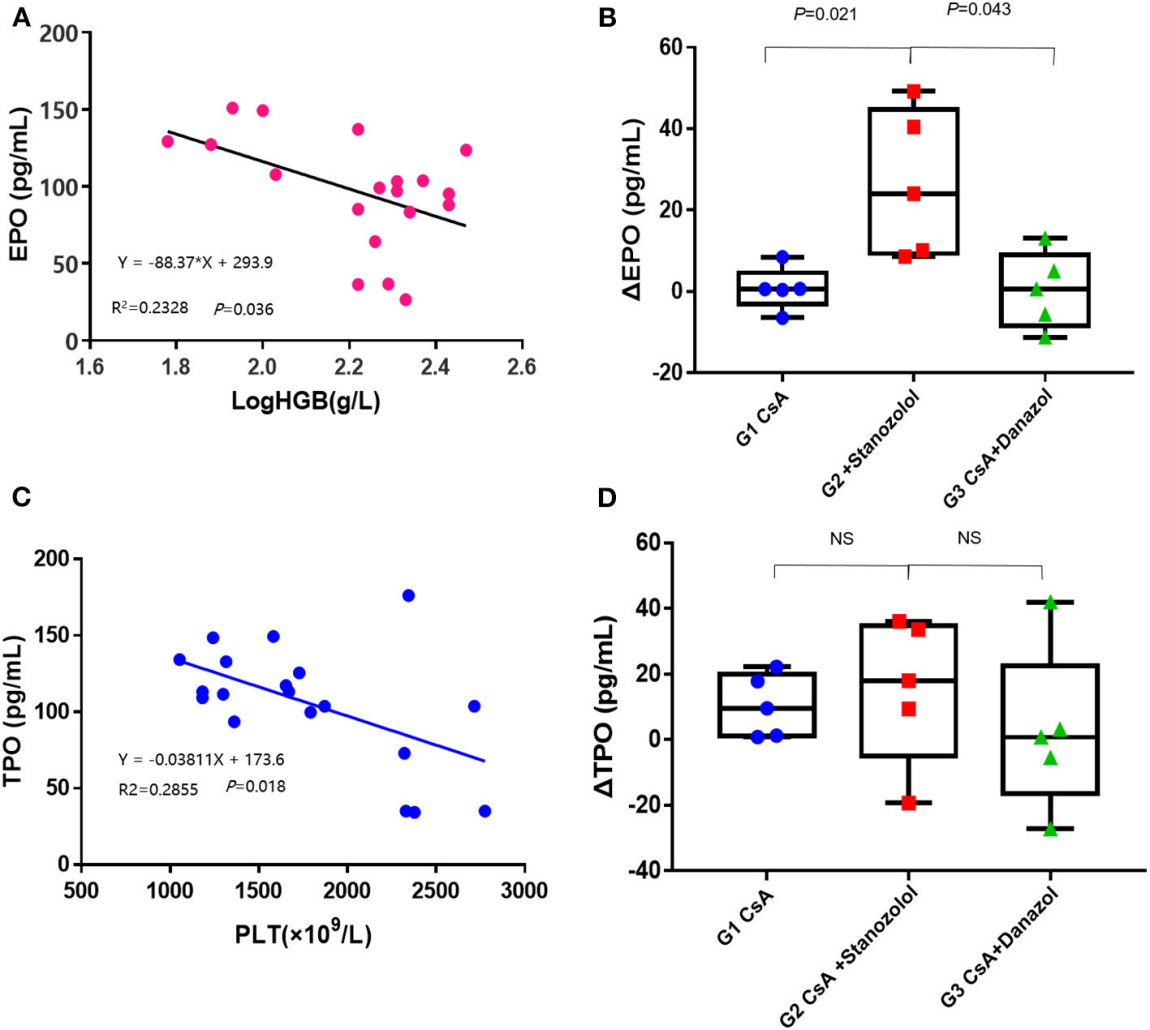
Serum levels of EPO and TPO in AA mice. **(A)** The EPO levels were negatively and linearly correlated with the log HGB levels. **(B)** The value of ΔEPO in mice treated with CsA+stanozolol was markedly higher than that in mice in the other two groups. **(C)** The level of TPO was negatively and linearly correlated with PLT counts. **(D)** The value of ΔTPO was not significantly different among the various treatment groups. G1, CsA; G2, CsA+Stanozolol; G3. CsA+Danazol. ΔEPO, difference between the actual EPO and expected EPO levels; ΔTPO difference between the actual and expected TPO levels; NS indicates *P* > 0.05.

The EPO levels were negatively and linearly correlated with Log HGB levels (Y = −88.37X+293.9, *r*^2^ = 0.2328, *P* = 0.036, Figure 6A). This formula was used to calculate the expected EPO level by adjusting the HGB level. Delta EPO (ΔEPO, the difference between the actual EPO level and expected EPO level) was compared among the various treatment groups, and the mice treated with CsA+ stanozolol exhibited higher ΔEPO levels than those treated with CsA+danazol and CsA alone (Figure 6B). Similarly, the TPO level was negatively and linearly correlated with the PLT level (Y = −0.03811X+173.6, *r*^2^ = 0.2855, *P* = 0.018). This formula was used to calculate the expected TPO level by adjusting the PLT level. However, there was no difference in ΔTPO (defined as the difference between the actual and expected TPO levels) among the various treatment groups (Figures 6C,D).

#### EPOR Expression in Bone Marrow Mononuclear Cells

**Figure 7:**
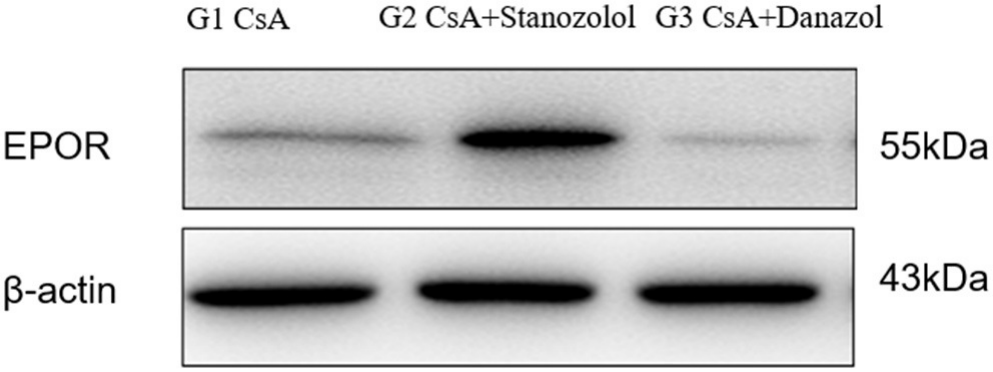
The expression of EPOR on bone marrow mononuclear cells in each treatment group, detected by western blotting. The figure showed results of one experiment.

The expression of EPOR was significantly higher in mice treated with CsA+stanozolol than that in the other two groups (Figure 7), indicating that stanozolol might improve erythroid hematopoiesis by upregulating the expression of EPOR.

## Discussion

In the middle of the last century, HGB levels were found to be generally higher in adult males than in females (11). In addition, animal experiments showed that castrated male mice exhibited decreased hematopoiesis and RBC count and that this effect can be markedly recovered using androgens (12). These observations indicate the potential role of androgens in stimulating hematopoiesis. In previous studies, the formation of erythroid colonies was dramatically improved by adding testosterone into the bone marrow culture system; it can be further enhanced using high concentrations of EPO (13). Testosterone can enhance the sensitivity of bone marrow precursor cells to EPO, exhibiting a synergistic effect with EPO (14). Androgens can elevate the serum EPO level in female mice and increase the level of HGB; this effect can disappear upon blocking EPO (15, 16). All these experiments were performed from 1940 through the 1960s, when knowledge regarding the mechanism of AA was limited.

Compared to testosterone, stanozolol and danazol are two synthetic steroid androgens with weaker androgen activities but protein assimilation effects. These androgens can facilitate the recovery of hematopoiesis in patients with AA (2, 17, 18). However, their mechanisms have not been fully illustrated, especially for stanozolol, which is not available in countries other than China.

In this study, we evaluated their effects on hematopoiesis using colony culture *in vitro* and in an immune-mediated AA mouse model *in vivo*. Our results suggested that neither stanozolol nor danazol exerted direct effects on the formation of bone marrow colonies, neither in patients with AA nor in normal controls. Similarly, no effect on erythroid or megakaryocyte differentiation was observed when K562 cells were incubated with the two drugs. Our results showed that the effects of stanozolol and danazol on hematopoiesis were different from that of natural androgens. Although weaker androgen activities might be part of the reason for this observation, these drugs may act through other molecules rather than acting directly on hematopoietic progenitor cells.

The immune-mediated AA mouse model can explain the actions of the two drugs more clearly. This animal model has been verified in many studies (19, 20). Our preliminary results in a mouse model of AA showed similar manifestations as that in patients with AA. In this animal model, stanozolol+CsA facilitated the recovery of erythroid hematopoiesis compared to CsA alone, as demonstrated by the HGB level and colony culture on day 30. CsA+danazol produced a quicker increase in PLT count than that obtained using CsA monotherapy. In addition, the combination of CsA with either stanozolol or danazol benefited WBC recovery compared to CsA monotherapy.

To further explain the underlying mechanisms of these drugs, we first analyzed their effects on immune dysfunction in AA mice. As shown in our animal model, a severely inverted proportion of CD4^+^/8^+^T cells and a decreased proportion of Treg/CD4^+^ cells was observed in the mouse model, suggesting a T cell dysfunction, which is one of the leading factors for the pathogenesis of AA (21–24). After treatment with CsA-based immunosuppression treatment, immune disorders were partially relieved. Both CsA+danazol and CsA+stanozolol demonstrated superior Treg recovery compared to CsA monotherapy, indicating that danazol and stanozolol might improve immune regulation. The immune regulatory effects of danazol were demonstrated by an increase in the proportion of Treg cells when administered alone in patients with acquired AA (25) and were further verified by Uchiyama et al., who showed that danazol monotherapy can prolong the survival of mice that underwent heart transplantation mice and elevate the proportion of spleen Treg cells (26). Our results in the animal model were consistent with these results. Meanwhile, we demonstrated the immune regulatory effects of stanozolol in our animal model, which has not been reported before.

Stimulation with TNFα and INFγ markedly increased Fas antigen expression in CD34^+^ cells, resulting in the transduction of a signal for cell death, leading to the failure of bone marrow hematopoiesis (27). Sloand et al. showed that INF-gamma increased in the peripheral blood lymphocytes of several patients with AA and declined with therapy. The presence of intracellular INF-gamma may predict response to immunosuppressive treatment and the onset of relapse (28, 29). It has been demonstrated that neutralizing donor T cell-derived TNFα *in vivo* increased short-term stem and progenitor cell engraftment, accelerated hematopoietic recovery, and altered donor immune cell compositions (30). In our study, serum inflammatory factors were evaluated to explore the immune-regulatory capacity of stanozolol and danazol. Our study showed that stanozolol and danazol can both suppress the expression of TNFα and upregulate the anti-inflammatory factor IL-10 and danazol can downregulate the levels of INFγ and IL-2, exhibiting a more comprehensive effect on immune regulation than stanozolol. As was shown in our *in vitro* experiments, stanozolol and danazol had no direct effects on hematopoietic progenitor cells. We thought they could partly restore hematopoiesis in AA mice through the inhibition of negative immune regulatory factors. Though we did not detect the relationship between the immune regulation and blood cell recovery directly, it has been demonstrated that improved immune status can help hematopoietic recovery, as shown by the effectiveness of IST in aplastic anemia (1, 31). Another direct and strong evidence was provided by Chen et al. (32) in their study on lymphocyte infusion-induced AA mouse model. In this study, AA mice were injected with Tregs through tail vein and significantly (*p* < 0.05) higher levels of residual BM cells and higher concentrations of white blood cells, RBCs, and platelets were observed compared to AA mice without Tregs injection. On the other hand, the immune regulatory effects of androgens may mediate by the macrophage polarization, as demonstrated by Lai et al. that androgen receptor was capable to control inflammatory response and regulate macrophage function (33). Macrophages were important in the pathogenesis of murine immune-mediated BM failure, as shown by Sun et al. that TNF-α from host macrophages and TNF-α R expressed on donor effector T cells of the mice were critical for the BM failure, acting by modulation of IFN-γ secretion. Similarly, in AA patients, TNF-α-producing macrophages in the BM were more frequent than in healthy controls, suggesting the involvement of this cytokine and these cells in human disease (34).

In addition to their immune-regulatory effects, which are not lineage specific, other mechanisms may exist for stanozolol and danazol because they showed effects in different lineages. The serum level of ΔEPO in mice treated with CsA+stanozolol was much higher than that in the other two groups, explaining the effect of stanozolol on erythroid hematopoiesis to some extent; this result was consistent with the results for testosterone (35, 36). Further examination showed that mice treated with CsA+stanozolol exhibited remarkably higher expression of EPOR in bone marrow mononuclear cells, indicating the effects of stanozolol on the secretion of EPO and EPOR. However, no such effects were observed for danazol; furthermore, no difference in the level of serum ΔTPO was found among different treatment groups, implying other potential mechanisms of action for danazol.

For the first time, the possible action of both stanozolol and danazol was investigated and compared using *in vitro* culture of bone marrow cells and *in vivo* experiments using an immune-mediated AA mouse model. Our results support the conclusion that both stanozolol and danazol influence hematopoiesis in AA; stanozolol is more effective for erythropoiesis through the EPO and EPOR pathways, and danazol is more effective for PLT recovery. Besides, they both have immunomodulatory effects; however, further studies exploring their mechanisms are indispensable.

## Data Availability Statement

The raw data supporting the conclusions of this article will be made available by the authors, without undue reservation.

## Ethics Statement

The studies involving human participants were reviewed and approved by the ethics committee of Peking Union Medical Colleague Hospital. The patients/participants provided their written informed consent to participate in this study. The animal study was reviewed and approved by the ethics committee of Peking Union Medical Colleague Hospital.

## Author Contributions

BH and HML designed the study and wrote the manuscript. HML conducted the experiments and processed the data. ZBL and TW provided experimental technical guidance and helped analyze experimental data. All authors reviewed the manuscript and approved the final submission of the manuscript.

## Conflict of Interest

The authors declare that the research was conducted in the absence of any commercial or financial relationships that could be construed as a potential conflict of interest.

## Supplementary Material

Supplementary Figure 1 Proportion of CD4+CD25+Foxp3+Treg cells in mice administered with different treatments. **(A)**. Proportion of CD4+CD25+Foxp3+Treg cells detected by flow cytometry. **(B)** Treg cells marked as CD4+ CD25+Foxp3+ cells in different groups. G1 CsA; G2 CsA+Stanozolol; G3 CsA+Danazol; G4 untreated; G5 normal control; ^*^indicates *P* < 0.05; NS indicates *P* > 0.05.
