# Supplementary figures and images for: Stanozolol and Danazol Have Different Effects on Hematopoiesis in the Murine Model of Immune-Mediated Bone Marrow Failure
Source: Front Med (Lausanne). 2021 May 28;8:615195. doi: 10.3389/fmed.2021.615195 (PMC8193361; doi:10.3389/fmed.2021.615195)

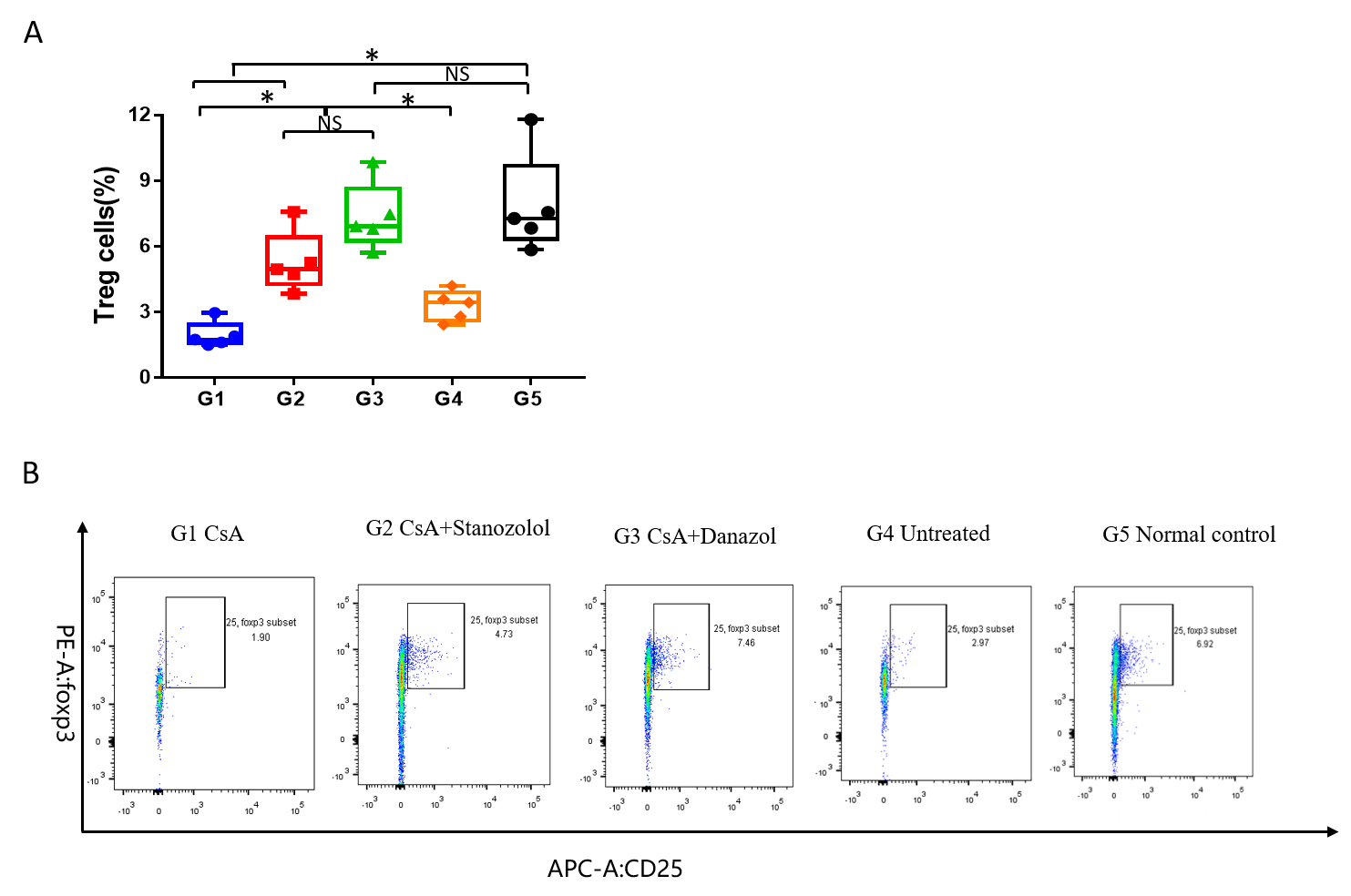

Supplement: Supplementary Figure 1 — Proportion of CD4+CD25+Foxp3+Treg cells in mice administered with different treatments. (A). Proportion of CD4+CD25+Foxp3+Treg cells detected by flow cytometry. (B) Treg cells marked as CD4+ CD25+Foxp3+ cells in different groups. G1 CsA; G2 CsA+Stanozolol; G3 CsA+Danazol; G4 untreated; G5 normal control; *indicates P < 0.05; NS indicates P > 0.05. [file Image_1.tif]
